# A scientometric visualization analysis of the gut microbiota and gestational diabetes mellitus

**DOI:** 10.3389/fmicb.2025.1485560

**Published:** 2025-01-30

**Authors:** Zehao Su, Lina Liu, Jian Zhang, Jingjing Guo, Guan Wang, Xiaoxi Zeng

**Affiliations:** ^1^West China Biomedical Big Data Center, West China Hospital, Sichuan University, Chengdu, Sichuan, China; ^2^Med-X Center for Informatics, Sichuan University, Chengdu, China; ^3^Center for Pathogen Research, West China Hospital, Sichuan University, Chengdu, China

**Keywords:** gut microbiota, gestational diabetes mellitus, bibliometric analysis, visualization, co-occurrence analysis

## Abstract

**Background:**

The prevalence of gestational diabetes mellitus (GDM), a condition that is widespread globally, is increasing. The relationship between the gut microbiota and GDM has been a subject of research for nearly two decades, yet there has been no bibliometric analysis of this correlation. This study aimed to use bibliometrics to explore the relationship between the gut microbiota and GDM, highlighting emerging trends and current research hotspots in this field.

**Results:**

A total of 394 papers were included in the analysis. China emerged as the preeminent nation in terms of the number of publications on the subject, with 128 papers (32.49%), whereas the United States had the most significant impact, with 4,874 citations. The University of Queensland emerged as the most prolific institution, contributing 18 publications. Marloes Dekker Nitert was the most active author with 16 publications, and Omry Koren garnered the most citations, totaling 154. The journal *Nutrients* published the most studies (28 publications, 7.11%), whereas *PLoS One* was the most commonly co-cited journal, with a total of 805 citations. With respect to keywords, research focuses can be divided into 4 clusters, namely, “the interrelationship between the gut microbiota and pregnancy, childbirth,” “the relationship between adverse metabolic outcomes and GDM,” “the gut microbiota composition and metabolic mechanisms” and “microbiota and ecological imbalance.” Key areas of focus include the interactions between the gut microbiota and individuals with GDM, as well as the formation and inheritance of the gut microbiota. Increasing attention has been given to the impact of probiotic supplementation on metabolism and pregnancy outcomes in GDM patients. Moreover, ongoing research is exploring the potential of the gut microbiota as a biomarker for GDM. These topics represent both current and future directions in this field.

**Conclusion:**

This study provides a comprehensive knowledge map of the gut microbiota and GDM, highlights key research areas, and outlines potential future directions.

## 1 Introduction

Gestational diabetes mellitus (GDM) is a glucose metabolism disorder that first manifests during pregnancy in women who have normal glucose metabolism prior to becoming pregnant (ref). In most pregnancies where it occurs, gestational diabetes mellitus (GDM) seems to result from an inadequate pancreatic response caused by the inability to counteract insulin resistance during pregnancy (Luo et al., 2023). The increasing prevalence of obesity and diabetes during pregnancy presents a significant public health challenge. These conditions not only increase the risk of cardiovascular disease among pregnant women but also exacerbate the prevalence of perinatal complications, including polyhydramnios, macrosomia, and neonatal asphyxia (Alejandro et al., 2020; Joergensen et al., 2014; Zhu and Zhang, 2016). Moreover, offspring of mothers with these conditions are at high risk of developing type 2 diabetes (Sears and Hivert, 2015), obesity (Page et al., 2014), metabolic syndrome (Boney et al., 2005), and other related chronic diseases, posing a significant threat to maternal and infant health (Heckbert et al., 1988).

Both maternal and child health are significantly impacted by GDM. The optimal prenatal care and the healthy development of mothers and children are vital to promoting the well-being of families and society as a whole. The acknowledged risk factors contributing to the increased incidence of diabetes include advanced age in mothers, prepregnancy overweight and obesity, excessive weight accumulation during pregnancy, insufficient physical activity, unhealthy dietary habits, racial background, and family history (Committee ACoOaG, 2018; Hod et al., 2015). In the field of diabetes management, there has been continuous interest in investigating both conventional drugs and natural herbal phytoconstituents (Roy et al., 2024). Owing to the distinctive nature of pregnancy, medication options for GDM patients are notably limited. Recently, researchers have focused on the connection between GDM and the gut microbiota. Targeted interventions based on the microbiota profile are being explored with the goal of offering innovative strategies for diagnosing, managing, and preventing GDM (Nazli et al., 2004).

Under normal conditions, the gut microbiota and the host maintain a delicate balance, coexisting in a harmonious and mutually beneficial symbiotic relationship (Huo et al., 2019). The gut microbiota contributes to energy metabolism, short-chain fatty acid production (Bäckhed et al., 2004), vitamin synthesis (Girdwood, 1955), the release of gastrointestinal hormones (Everard and Cani, 2014), the preservation of gut barrier integrity (Plöger et al., 2012), and the activation of the immune system through the digestion and breakdown of food in the intestines (Kau et al., 2011). Disruptions in the intestinal ecosystem can affect this symbiotic relationship, potentially through factors such as obesity, aging, dietary habits, and sedentary lifestyles. Additionally, antibiotics can change the makeup or configuration of the gut microbiota (Lloyd-Price et al., 2019).

Metabolism during a typical pregnancy is a dynamic and intricately regulated process. In contrast to nonpregnant periods, pregnancy induces notable metabolic changes, including shifts in women’s serum fatty acid and amino acid levels (Lloyd-Price et al., 2019). With increasing gestational age, these changes can lead to variations in the gut microbiota (Feng et al., 2020; Fu et al., 2020). Research indicates that, during late pregnancy, women tend to possess increased relative abundances of *Proteobacteria* and *Actinobacteria* (Koren et al., 2012). Additionally, pregnant women diagnosed with GDM typically present a decreased proportion of *Firmicutes* (Fugmann et al., 2015), a finding also observed in research on type 2 diabetes (Karlsson et al., 2013). Research also indicates that the microbiota in a pregnant woman’s body can impact pregnancy outcomes (Shiozaki et al., 2014). Disruption of the gut microbiota can lead to abnormalities in the metabolism of glucose and lipids in host cells. It can also cause dysregulation of inflammatory cytokine expression (Joshi et al., 2024). This dysregulation exacerbates preexisting physiological conditions, such as insulin resistance and hyperlipidemia. Consequently, it promotes adverse pregnancy outcomes (Lundgren et al., 2018).

Understanding the relationship between the gut microbiota and GDM is critical for improving the prevention and treatment of GDM. It also plays an important role in ensuring the health of mothers and their offspring. In recent years, this field has attracted increasing attention from researchers and clinicians, leading to many important findings. It is essential to systematically review the relationship between gut microbiota and GDM. Based on preliminary literature research, bibliometric methods have already been applied to conduct systematic reviews on the relationship between gut microbiota and type 1 or type 2 diabetes (Guo et al., 2023; Jiang et al., 2024). This method applies specialized visualization tools to map research networks, knowledge structures, and emerging trends. Compared with traditional reviews, bibliometric analysis includes quantitative methods. It offers a multidimensional view of the field by identifying research hotspots and potential future directions. This study also employs bibliometric methods, aiming to summarize the research progress and key issues concerning the relationship comprehensively and accurately between gut microbiota and GDM. The findings are intended to assist researchers in tracking developments in gut microbiota and GDM research and identifying potential opportunities for further exploration.

## 2 Methods

### 2.1 Sources of data and search methodologies

**Figure 1:**
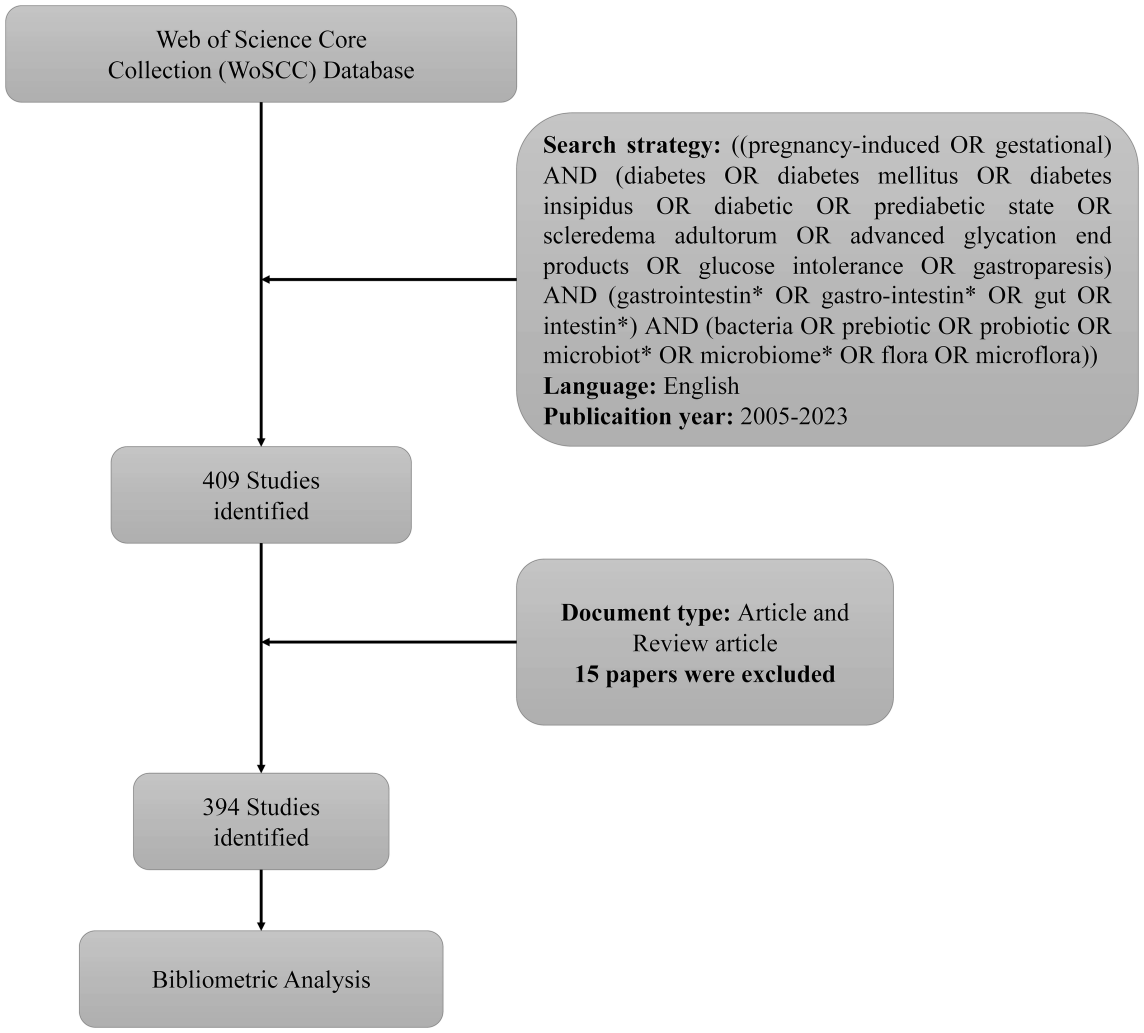
Flow diagram of the inclusion process.

Data for bibliographic analysis were retrieved from the Core Collection of Web of Science (WoSCC), a Clarivate Analytics database known for its high-quality literature. The following search strategies were used: [(gestational OR pregnancy-induced) AND (diabetes mellitus OR diabetes OR diabetic OR diabetes insipidus OR prediabetic state OR scleredema adultorum OR advanced glycation end products OR gastroparesis OR glucose intolerance) AND (intestin* OR gut OR gastrointestin* OR gastro-intestin*) AND (microflora OR probiotic OR prebiotic OR microbiome* OR microbiot* OR flora OR bacteria)]. Only articles written in English were selected. All records were downloaded in the “Full Record and Cited References” format and saved as “plain text files” and “Bibtex.” To prevent any bias from daily database updates, all the data were collected on January 13, 2024 (Figure 1).

### 2.2 Data analysis and graph acquisition

The documents procured from WoSCC were transferred into Microsoft Excel [version 16.66.1], VOSviewer [version 1.6.19], and CiteSpace [version 6.1. R6] for bibliometric and visual analysis. Microsoft Excel was used to analyze annual publication trends and publisher outputs. VOSviewer was employed to conduct visual analyses of collaborative networks involving countries, institutions, journals, authors, and the co-occurrence of keywords. Both the analysis of the citation bursts of references and the creation of dual map overlays were expertly handled via CiteSpace. The Biblioshiny platform offers a web graphical user interface for Bibliometrix to create country collaboration maps. Impact factor (IF) data were sourced from the 2022 Journal Citation Reports.

## 3 Results

### 3.1 Overview of the yearly growth trajectory

**Figure 2:**
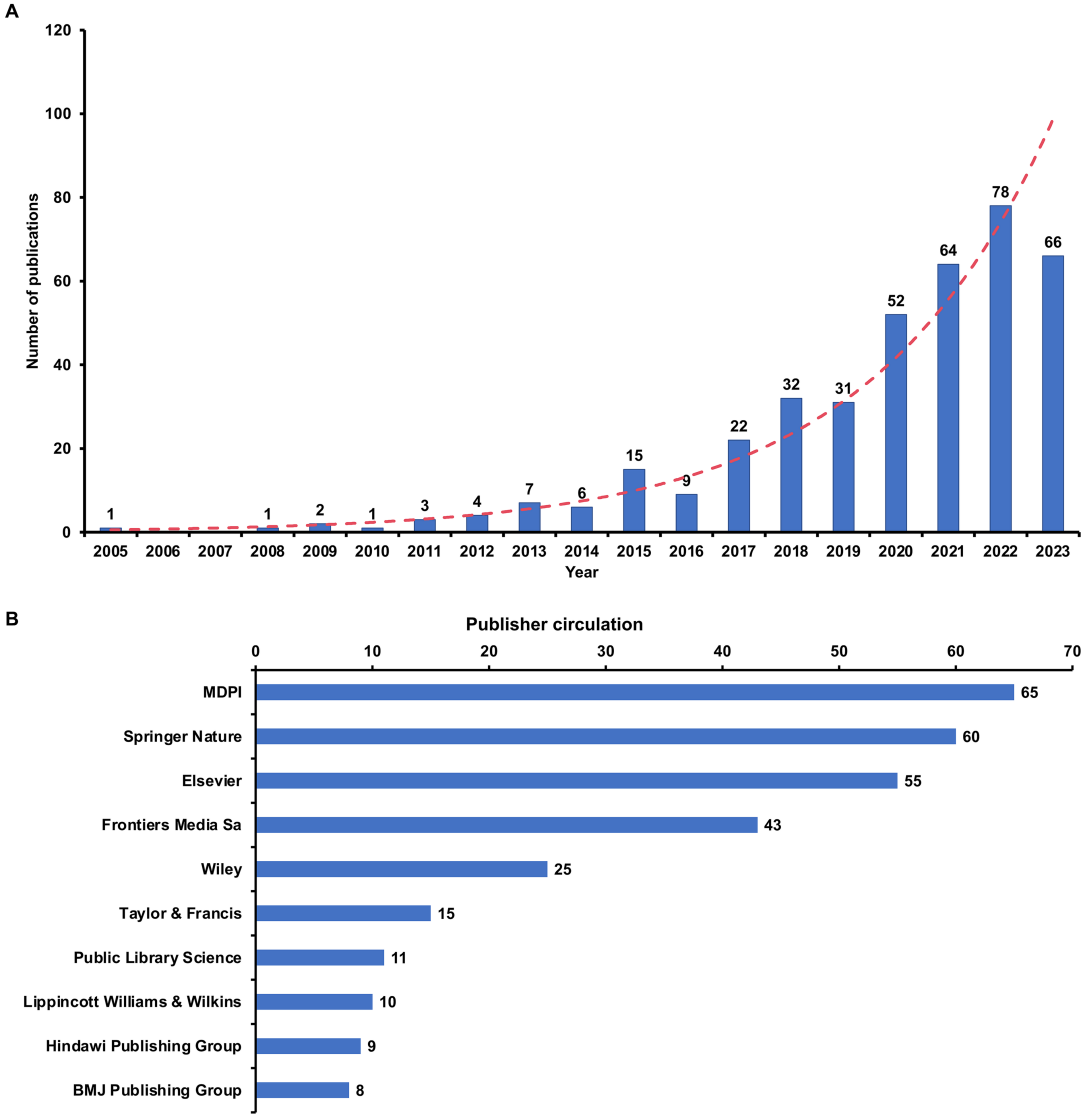
Global publication trends for gut microbiota and GDM research. **(A)** Annual global publication output; **(B)** Top 10 publishers on the basis of their individual contributions to the overall article count of the gut microbiota and GDM research.

The analysis included 394 papers, consisting of 262 research articles and 132 review articles on the gut microbiota and GDM. As shown in Figure 2A, there was an overall upward trajectory in publication volume on this topic from 2005, when the first article was published, to 2023. Figure 2B highlights the top 10 publishers that significantly contributed to the overall number of articles concerning the gut microbiota and GDM. The MDPI (*n* = 65), Springer Nature (*n* = 60), and Elsevier (*n* = 55) led the way, significantly outpacing other publishers and reaffirming their dominant international position within the realm of publishing.

### 3.2 Countries/regions influence and collaboration

**Figure 3:**
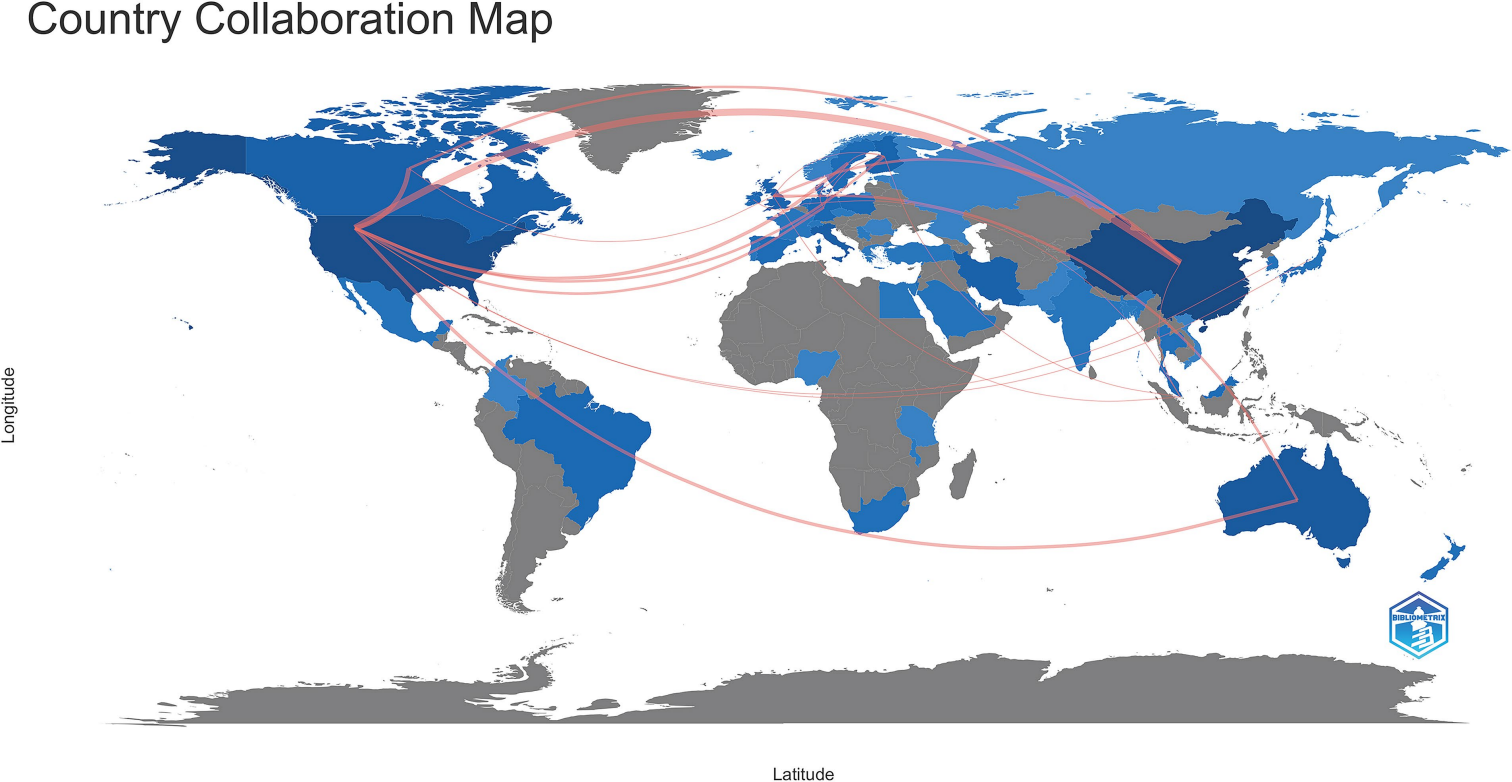
A comprehensive global visualization map showing publications and collaborative networks. The intensity of the blue shading signifies the quantity of articles, with darker blue indicating a greater number of publications. The red lines represent academic collaborations between connected countries, with thicker lines indicating stronger cooperation.

From 2005--2023, a total of 50 countries/regions engaged in research pertaining to the interplay between the gut microbiota and GDM. The top 5 countries with respect to publication numbers were China, with 128 papers (32.49% of the total), followed by the United States with 93 papers (23.60%), Australia with 36 papers (9.14%), Italy with 23 papers (5.84%), and Canada with 19 papers (4.82%) (Table 1). However, the United States had the most citations (4,874), followed by Finland (2,657), China (2,300), and Australia (2,163). Sweden, with 8 publications, recorded the greatest average number of citations per article (193.00), reflecting exceptionally high research quality. Similar trends were observed in several European countries, such as Finland (average number of citations per article, 166.06) and the Netherlands (average number of citations per article, 124.00) (Table 2). The country collaboration map provides an overview of global academic cooperation (Figure 3). The United States boasted the most extensive academic connections with various countries/regions, where China represented the closest collaboration, followed by Canada and Australia.

**Table 1:** Top 10 most prolific countries/regions in research pertaining to the gut microbiota and GDM research.

Rank	Countries	Articles	Percentage (N/394)
1	China	128	32.49%
2	The United States	93	23.60%
3	Australia	36	9.14%
4	Italy	23	5.84%
5	Canada	19	4.82%
6	Iran	18	4.57%
7	Finland	16	4.06%
8	England	15	3.81%
9	Poland	11	2.79%
10	Germany	10	2.54%

**Table 2:** The top 10 countries/regions with the highest local citation rates in research on the gut microbiota and GDM research.

Rank	Countries	Total citations	Citations per article
1	The United States	4,874	52.41
2	Finland	2,657	**166.06**
3	China	2,300	17.97
4	Australia	2,163	60.08
5	Italy	1,915	83.26
6	Sweden	1,544	**193.00**
7	England	802	53.47
8	Netherlands	744	**124.00**
9	Canada	733	38.58
10	Singapore	723	120.50

The bold values indicate the top three countries/regions in terms of citations per article.

### 3.3 Contributions of different institutions

**Figure 4:**
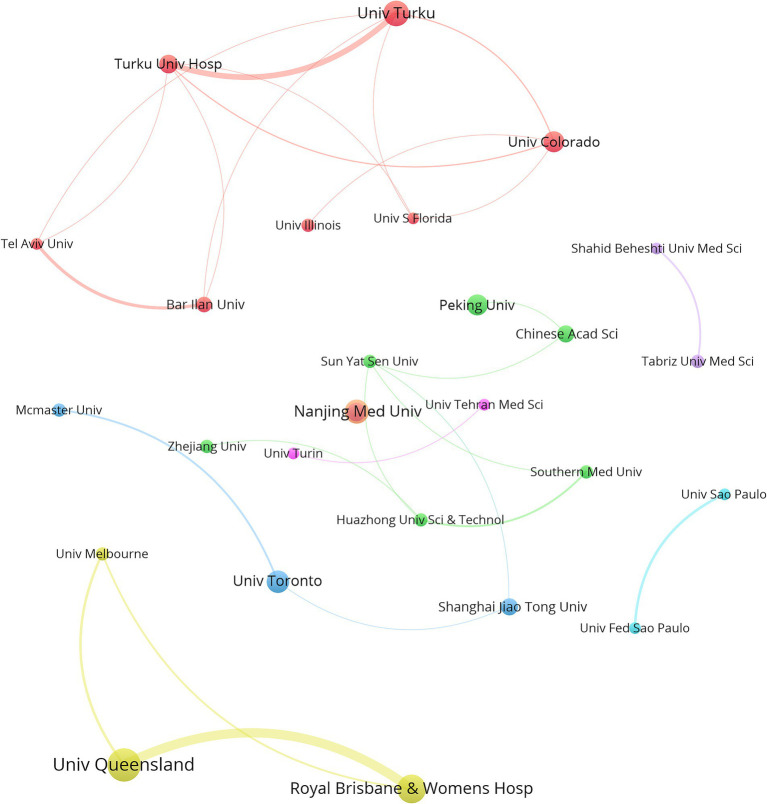
VOSviewer network visualization highlights the collaborative efforts of institutions engaged in research on the gut microbiota and GDM. The magnitude of the nodes signifies the quantity of publications, with larger nodes indicating more publications, and the width of the lines denotes the intensity of collaboration between institutions. The Minimum number of documents of an organization is 5. Normalization Method: LinLog/modularity, Layout Attraction: 9, Layout Repulsion: -2.

In total, 744 institutions were included in the institutional analysis. The 10 institutions with the most significant publication outputs are listed in Table 3. The University of Queensland led with 18 papers, followed by the Royal Brisbane and Women’s Hospital with 15 papers and the University of Turku with 13 papers. Four of the top 10 institutions are headquartered in China, whereas Australia and Finland each have two institutions in the top 10. The University of Turku recorded the greatest number of citations (2,009), followed by the University of Colorado (1,716). Notably, Turku University Hospital published only 9 articles but ranked third in citations, with 1,669 (Table 3). Figure 4, generated via VOSviewer, illustrates the collaboration between institutions, highlighting the University of Queensland in Australia’s strong focus on the gut microbiota and GDM research.

**Table 3:** The top 10 leading institutions with the greatest number of publications in the field of gut microbiota and GDM research.

Rank	Institutions	Articles	Citations	Country
1	The University of Queensland	18	1,190	Australia
2	Royal Brisbane & Women’s Hospital	15	1,147	China
3	University of Turku	13	**2,009**	Finland
4	Nanjing Medical University	12	249	Australia
5	University of Toronto	11	471	Finland
6	University of Colorado	10	**1,716**	China
7	Peking University	10	61	China
8	Turku University Hospital	9	**1,669**	Canada
9	Chinese Academy of Medical Sciences	9	270	China
10	Chinese Academy of Sciences	8	297	Israel

The bold values indicate the top three institutions in terms of total article citations.

### 3.4 Distribution and co-authorship of authors

**Figure 5:**
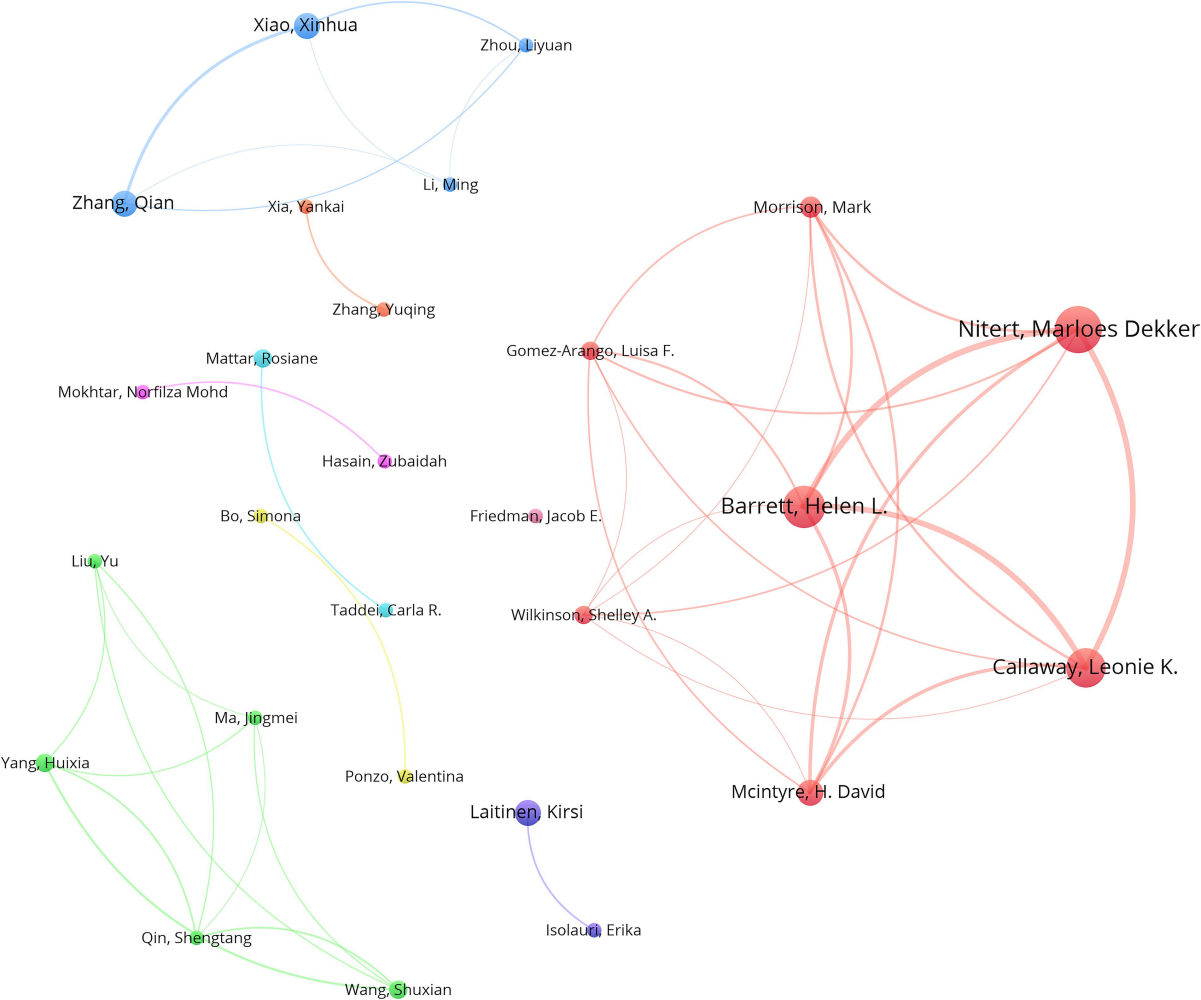
VOSviewer network visualization portrays the connections between authors active in the gut microbiota and GDM research. The node sizes are indicative of the quantity of publications, with larger nodes indicating more publications, and the width of the lines denotes the intensity of collaboration between authors. The Minimum number of documents of an author is 4. Normalization Method: LinLog/modularity, Layout Attraction: 9, Layout Repulsion: -2.

The analysis of the included articles revealed that research focusing on the gut microbiota and GDM was conducted by 2,338 researchers. Marloes Dekker Nitert, hailing from the University of Queensland in Australia, topped the list, with an impressive total of 16 publications. Helen L. Barrett (14 papers) is affiliated with the University of Queensland, Australia, whereas Leonie K. Callaway (13 papers) is also affiliated with the University of Queensland, Australia (Table 4). VOSviewer was used to analyze author information and explore academic relationships between authors (Figure 5). Omry Koren from Bar-Ilan University, Israel, led with 154 co-cited articles, followed by Mie Korslund Wiinblad Crusell (131 co-cited articles) from the University of Copenhagen, Denmark, and Boyd E. Metzger (120 co-cited articles) from the Northwestern University Feinberg School of Medicine, Chicago, IL, United States. Table 5 presents the top 10 most frequently co-cited authors.

**Table 4:** Top 10 most productive authors related to the gut microbiota and GDM research.

Rank	Authors	Articles	Country	Institutions
1	Marloes Dekker Nitert	16	Australia	The University of Queensland
2	Helen L. Barrett	14	Australia	The University of Queensland
3	Leonie K. Callaway	13	Australia	The University of Queensland
4	Kirsi Laitinen	8	Finland	University of Turku
5	David H. Mcintyre	8	Australia	The University of Queensland
6	Xinhua Xiao	8	China	Peking Union Medical College
7	Qian Zhang	8	China	Peking Union Medical College
8	Mark Morrison	6	Australia	The University of Queensland
9	Luisa F. Gomez-Arango	5	Australia	The University of Queensland
10	Shelley A. Wilkinson	5	Australia	The University of Queensland

**Table 5:** Top 10 co-cited authors related to the gut microbiota and GDM research.

Rank	Cited authors	Articles	Country	Institutions
1	Omry Koren	154	Israel	Cornell University
2	Mie Korslund Wiinblad Crusell	131	Denmark	University of Copenhagen
3	Boyd E. Metzger	120	The United States	Northwestern University
4	Maria Carmen Collado	119	Spain	Spanish National Research Council
5	Luisa F. Gomez-arango	97	Australia	The University of Queensland
6	Patrice D. Cani	96	Belgium	Université Catholique de Louvain
7	Ya-Shu Kuang	91	China	Guangzhou Medical University
8	Jinfeng Wang	90	China	Chinese Academy of Sciences
9	Kati Mokkala	88	Finland	University of Turku
10	Peter J Turnbaugh	88	The United States	University of California

### 3.5 Analysis of journals and co-cited academic journals

Among the academic journals publishing articles on the gut microbiota and GDM, *Nutrients* (28 papers, 7.11%, IF 2022 = 5.90) ranked first, followed by *Frontiers in Microbiology* (12 papers, 3.05%, IF 2022 = 5.20), *Frontiers in Endocrinology* (10 papers, 2.54%, IF 2022 = 5.20), and *PLoS One* (10 papers, 2.54%, IF 2022 = 3.70), both tied for third place. Table 6 lists the top 10 journals by publication count, with 40% of the journals from Switzerland, 40% from the United States, and 20% from England. The top-ranked journal in 2022 by impact factor was *Gut Microbes* (IF 2022 = 12.20) from the United States. *PLoS One* (IF 2022 = 3.70) led with a total of 805 citations, followed by *Diabetes Care* (IF 2022 = 16.20) with 787 citations and *Nature* (IF 2022 = 64.80) with 590 citations (Table 7). Among the prestigious top 10 influential journals in gut microbiota and GDM research, three are also among the top 10 co-cited journals (*Nutrients*, *PLoS One*, *Scientific Reports*). The dual map overlay analysis depicted in Supplementary Figure S1 identifies five primary citation pathways, distinguished by orange or green colors.

**Table 6:** Top 10 most productive journals in the field of gut microbiota and GDM research.

Rank	Journal	Articles	Percentage (N/394)	Country	IF (2022)
1	*Nutrients*	28	7.11%	Switzerland	5.90
2	*Frontiers in Microbiology*	12	3.05%	Switzerland	5.20
3	*Frontiers in Endocrinology*	10	2.54%	the United States	5.20
4	*PLoS One*	10	2.54%	the United States	3.70
5	*BMC Pregnancy and Childbirth*	7	1.78%	England	3.10
6	*Microorganisms*	7	1.78%	Switzerland	4.50
7	*Scientific Reports*	7	1.78%	England	4.60
8	*Frontiers in Cellular and Infection Microbiology*	6	1.52%	Switzerland	5.70
9	*Gut Microbes*	6	1.52%	The United States	12.20
10	*International Journal of Molecular Sciences*	6	1.52%	The United States	5.60

**Table 7:** Top 10 co-cited journals in the field of gut microbiota and GDM research.

Rank	Co-cited journal	Citation	Country	IF (2022)
1	*PLoS One*	805	The United States	3.70
2	*Diabetes Care*	787	The United States	16.20
3	*Nature*	590	England	64.80
4	*Nutrients*	567	Switzerland	5.90
5	*Scientific Reports*	506	England	4.60
6	*American Journal of Clinical Nutrition*	442	The United States	7.10
7	*Diabetes*	430	The United States	7.70
8	*British Journal of Nutrition*	395	England	3.60
9	*Proceedings of the national academy of sciences (PNAS)*	368	The United States	11.10
10	*American Journal of Obstetrics and Gynecology*	362	The United States	9.80

### 3.6 Analysis of keywords

**Figure 6:**
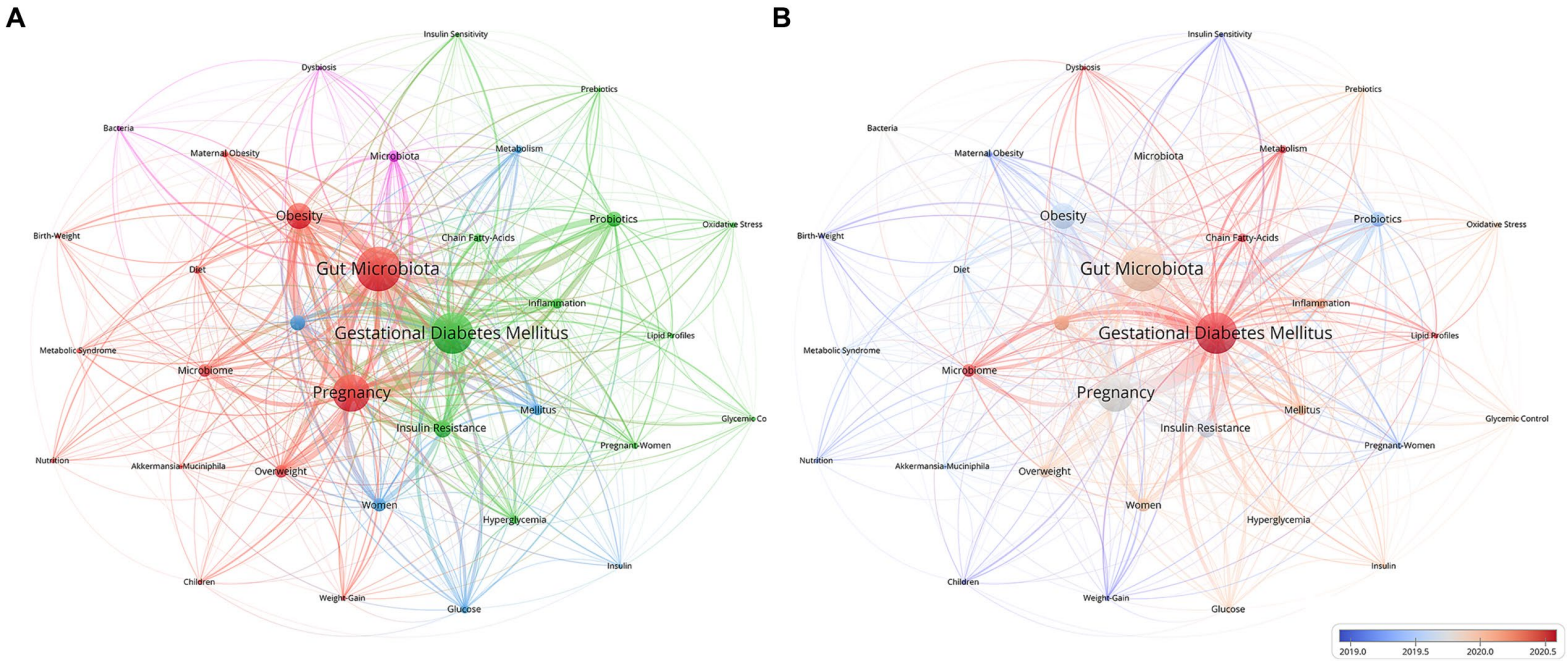
Keywords co-occurrence network for the gut microbiota and GDM research. **(A)** Network visualization of keywords via VOSviewer. **(B)** Overlay of keywords also visualized with VOSviewer. In the map, the node size mirrors the term usage frequency across publications, whereas the connecting lines represent the relationships between terms. Larger nodes indicate more frequent usage of a term, and thicker lines signify stronger relationships. The Minimum number of documents of a keyword is 13. Normalization Method: Association Strength, Layout Attraction: 3, Layout Repulsion: -1.

As shown in Figure 6A, “gut microbiota,” “gestational diabetes mellitus,” “pregnancy,” “obesity,” and “gut microbiome” constitute the primary research framework in this field. All keywords were clustered into four categories. The newest salient keywords were “gestational diabetes mellitus,” “microbiome,” “chain fatty acids,” “metabolism,” “lipid profiles,” and “dysbiosis,” which were identified after 2020, indicating that this term has been the focus in recent years (Figure 6B).

### 3.7 Analysis of co-cited references and reference burst detection

Table 8 shows the top 10 most frequently co-cited references related to the gut microbiota and GDM. The most frequently cited publications were from 2008--2019, with six studies published after 2016. The most cited paper was authored by Koren et al. (2012) and published in *Cell*, with 150 citations. The second most cited article, published by Crusell et al. (2018) in *Microbiome*, received 113 citations. The third most cited paper, authored by Kuang et al. (2017) and published in *Gigascience*, had 91 citations. Highlighted in Table 9, the 25 foremost references, distinguished by their exceptional citation surges, serve as pivotal milestones, significantly influencing and guiding the field’s evolution over an extended period.

**Table 8:** The top 10 co-cited references related to the gut microbiota and GDM research.

Rank	Title	Author	Article type	Journal	DOI	Articles
1	Host remodeling of the gut microbiome and metabolic changes during pregnancy	Koren et al. (2012)	Research Article	*Cell*	10.1016/j.cell.2012.07.008	150
2	Gestational diabetes is associated with change in the gut microbiota composition in third trimester of pregnancy and postpartum	Crusell et al. (2018)	Research Article	*Microbiome*	10.1186/s40168-018-0472-x	113
3	Connections between the human gut microbiome and gestational diabetes mellitus	Kuang et al. (2017)	Research Article	*Gigascience*	10.1093/gigascience/gix058	91
4	Dysbiosis of maternal and neonatal microbiota associated with gestational diabetes mellitus	Wang et al. (2018)	Research Article	*Gut*	10.1136/gutjnl-2018-315988	84
5	Changes in the gut microbiota composition during pregnancy in patients with gestational diabetes mellitus (GDM)	Ferrocino et al. (2018)	Research Article	*Scientific Reports*	10.1038/s41598-018-30735-9	75
6	Distinct composition of gut microbiota during pregnancy in overweight and normal-weight women	Collado et al. (2008)	Research Article	*American Journal of Clinical Nutrition*	10.1093/ajcn/88.4.894	61
7	A metagenome-wide association study of gut microbiota in type 2 diabetes	Qin et al. (2012)	Research Article	*Nature*	10.1038/nature11450	57
8	International association of diabetes and pregnancy study groups recommendations on the diagnosis and classification of hyperglycemia in pregnancy	Metzger et al. (2010)	Review Article	*Diabetes Care*	10.2337/dc09-1848	55
9	Microbiome and its relation to gestational diabetes	Cortez et al. (2019)	Research Article	*Endocrine*	10.1007/s12020-018-1813-z	53
10	Connections between the gut microbiome and metabolic hormones in early pregnancy in overweight and obese women	Gomez-Arango et al. (2016)	Research Article	*Diabetes*	10.2337/db16-0278	51

**Table 9:** Top 25 burst references in publications focused on the gut microbiota and GDM research.

References	Year	Strength	Begin	End	2005–2023
Laitinen et al. (2009)	2009	5.08	**2010**	2014	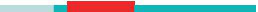
Luoto et al. (2010)	2010	6.77	**2012**	2015	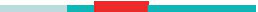
Koren et al. (2012)	2012	10.61	**2015**	2017	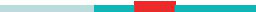
Vrieze et al. (2012)	2012	4.11	**2015**	2017	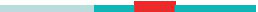
Qin et al. (2012)	2012	4.11	**2015**	2017	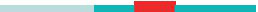
Nitert et al. (2013)	2013	6.51	**2014**	2018	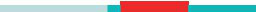
Asemi et al. (2013)	2013	5	**2014**	2018	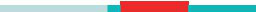
Aagaard et al. (2014)	2014	8.4	**2015**	2019	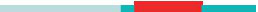
Lindsay et al. (2014)	2014	7.07	**2015**	2019	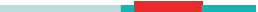
Wickens et al. (2017)	2017	4.54	**2018**	2019	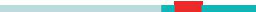
Ma et al. (2014)	2014	4.41	**2015**	2019	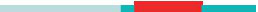
Barrett et al. (2014)	2014	4.25	**2014**	2019	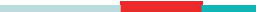
Fugmann et al. (2015)	2015	8.26	**2017**	2020	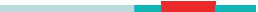
DiGiulio et al. (2015)	2015	8.26	**2017**	2020	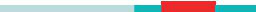
Dolatkhah et al. (2015)	2015	7.86	**2017**	2020	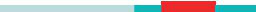
Lindsay et al. (2015)	2015	7.47	**2017**	2020	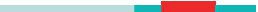
Gohir et al. (2015)	2015	5.49	**2017**	2020	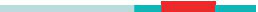
Bäckhed et al. (2004)	2015	4.7	**2017**	2020	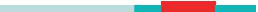
Bassols et al. (2016)	2016	4.1	**2017**	2020	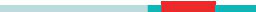
Damm et al. (2016)	2016	6.7	**2019**	2021	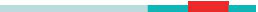
Gomez-Arango et al. (2016)	2016	6.23	**2017**	2021	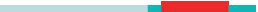
Zhu and Zhang (2016)	2016	6.14	**2020**	2021	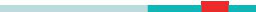
Karamali et al. (2016)	2016	5.79	**2018**	2021	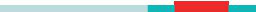
Jafarnejad et al. (2016)	2016	4.34	**2019**	2021	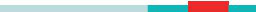
Kuang et al. (2017)	2017	4.77	**2020**	2023	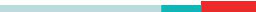

The blue bars represent the time intervals, with dark blue indicating the publication dates, and the red bars visually depict the citation bursts of the references. The bold values indicate the starting year of the citation burst. Time slicing: 2005--2023, with 1 year per slice, g-index: k = 25, pruning: pathfinder & pruning sliced networks, burstness: γ = 1.0, minimum duration = 2.

## 4 Discussion

This study employed bibliometrics and visual analytics to conduct a comprehensive analysis of the gut microbiota and GDM. The findings reveal an increasing number of scientific publications in this area in recent years, underscoring the growing importance of the field. Furthermore, this study enhances our understanding of research on the gut microbiota and GDM while highlighting opportunities for further investigation.

### 4.1 General information

Initially, the annual publication count from 2005--2010 remained somewhat stable with minimal fluctuation. However, after 2010, there was a significant surge in yearly publications, likely due to the emergence of numerous studies in this field driven by advancements in scientific and technological research. The extent of collaboration among countries/regions, institutions, and authors was evaluated. This assessment seeks to identify patterns in scientific research collaboration, offering insights to increase research efforts and identify potential collaboration opportunities among various groups.

As per Table 1, China ranks first in terms of the total number of publications, while the cumulative number of citations for publications from the United States is the highest. Both countries are clearly leaders in this field. Despite having fewer publications, Sweden, Finland, and the Netherlands have the most substantial average citations per article, indicating exceptionally high research quality in these countries and setting a benchmark for other regions within this research area (Table 2). Among the top 10 institutions, four are located in China, highlighting the country’s regional strengths and dominance in the field. This presence provides insight into China’s consistently high volume of publications. The University of Queensland, Australia, emerged as the world’s most prolific institution, followed by Royal Brisbane and Women’s Hospital, indicating their vast global partnerships with other institutions. Despite Italy and Iran ranking fourth and sixth in total publications, respectively, no research institutions from either country are among the top 10. This suggests a deficiency of institutions with considerable professional value and research standing in these two countries (Table 1, Table 3). Through close collaboration among different countries (Figure 3) and institutions, breakthroughs in this field are expected to continue overcoming existing challenges.

In the fields of the gut microbiota and GDM, Marloes Dekker Nitert, Helen L. Barrett, and Leonie K. Callaway have emerged as the top three authors with the greatest number of publications. Intriguingly, they all originate from the University of Queensland in Australia, further underscoring the institution’s pivotal position in this research domain. This clustering of top researchers from the University of Queensland reaffirms the university’s central role in advancing research in this field.

The scale of yearly scientific output is an essential marker of academic progress. *Nutrients* stands out as the leading journal with the highest volume of publications in this field, highlighting its significant contribution to gut microbiota and GDM research. The findings underscore its pivotal role in advancing knowledge and fostering dialog within the scientific community.

Keywords pinpoint the central topics and content of research. Analyzing the co-occurrence of keywords reveals the dissemination and evolution of research topics within a field. Through keyword clustering, the keywords can be divided into four clusters, with each cluster representing a different research direction: cluster #1, “the interrelationship between gut microbiota and pregnancy, childbirth” (color in red), which contains 13 keywords; cluster #2, “the relationship between adverse metabolic outcomes and GDM” (color in green), which contains 12 keywords; cluster #3, “the gut microbiota composition and metabolic mechanisms” (color in blue), which contains 6 keywords; and cluster #4, “microbiota and ecological imbalance” (color in yellow) (Figure 6A). Overlay visualization (Figure 6B) reveals emerging keywords, offering a distinct comprehension of cutting-edge research on the gut microbiota and its connection to GDM. The complex interplay between GDM and metabolism, the microbiome, and dysbiosis constitutes the forefront of current research. This insight underscores the intricate connections and potential avenues for further exploration in elucidating the pathophysiology and management of GDM.

Additionally burst detection is an effective analytical tool. It identifies references with significant citation bursts

which are recognized as critical milestones. These references often guide the development direction of the field for some time. By analyzing co-cited references keywords and burst references valuable insights can be gained into current trends and popular research areas (Figure 6; Tables 8, 9). These insights can be broadly categorized into four main aspects each highlighting pivotal aspects of gut microbiota and GDM research

### 4.2 The research hotspots and trending

#### 4.2.1 Interaction between the gut microbiota and the host

The gut microbiota is fundamental to host health, and research on metabolism and microbiota functionality has been steadily increasing over the years. This expanding field of research aims to elucidate the intricate interplay between the gut microbiota and their hosts. These findings shed light on how this relationship impacts various aspects of health and disease.

The composition of the gut microbiota can influence the host. In one study, lean donors provided fecal microbiota transplants to male patients suffering from metabolic syndrome. Following transplantation, the recipients exhibit improved insulin sensitivity and variations in their gut microbial community (Vrieze et al., 2012). A study of pregnant women with gestational diabetes revealed that the transfer of the microbiota from the third trimester (T3) into germ-free mice led to greater obesity and insulin resistance than did the microbiota from the first trimester (T1) (Koren et al., 2012). Notable differences were observed in BMI, Homeostatic Model Assessment of Insulin Resistance (HOMA-IR), fasting glucose levels, C-peptide, insulin, Gastrointestinal Polypeptide (GIP), leptin, and resistin levels were detected between pregnant women who were overweight or obese and those with normal weight. Specifically, obese women exhibit a more disturbed metabolic profile than overweight women do (Gomez-Arango et al., 2016). These studies indicate that the gut microbiota plays a crucial role in the metabolic health of different populations.

The host influences the gut microbiota, which in turn affects the host itself. There is a significant correlation between changes in the maternal gut microbiota during pregnancy and the dietary patterns of mothers before and during pregnancy. In one study, 26 genera of the gut microbiota significantly differed between control mice and those fed a high-fat diet during pregnancy. The high-fat diet group had a predominance of *Firmicutes*, especially within the *Clostridiales* order, which led to an increased *Firmicutes* to *Bacteroidetes* ratio. Importantly, these microbiota shifts in high-fat diet-fed pregnant mice were linked to pathways associated with lipid metabolism, glycolysis, and gluconeogenesis (Gohir et al., 2015). 16S rRNA sequencing of the fecal microbiota of GDM patients revealed a notable increase in *α* diversity, characterized by increased *Firmicutes* and reduced *Bacteroidetes* and *Actinobacteria* (Ferrocino et al., 2018). These findings are similar to the outcomes observed with a high-fat diet, suggesting that dietary habits during pregnancy may be contributing factors to GDM. This factor influences the proportion of the gut microbiota, which in turn can lead to metabolic abnormalities in pregnant women. Therefore, modifying dietary structure has the potential to be an effective measure for controlling GDM.

Many studies have consistently underscored the strong association between maternal plasma glucose concentrations during gestation and the occurrence of unfavorable pregnancy outcomes (Metzger et al., 2010; Pettitt et al., 1980; Sermer et al., 1995). Understanding the interactions between the gut microbiota and host metabolism is crucial. Targeted dietary interventions or other measures to alter the gut microbiota can significantly improve metabolic health and pregnancy outcomes.

#### 4.2.2 Generation and heredity of the microbial flora

After birth, newborns encounter a diverse array of microbes, many of which are transferred from the mother during and after delivery. This process establishes an ecosystem in infancy that is initially populated by a constrained assortment of bacterial taxa (Hyman et al., 2005; Zhou et al., 2007). Initial microbial exposure plays a pivotal role in shaping the developmental trajectory of the gut microbiota, fostering a more intricate and resilient adult microbial ecosystem. Early microbial communities can act as direct sources of both protective and pathogenic bacteria in infancy (Biasucci et al., 2008; Connell and Slatyer, 1977). The data analysis revealed distinct characteristics of early-life microbiota composition and ecological networks at each stage. The results also revealed that infants born via cesarean section had significantly lower similarity to their mothers’ microbiota than did vaginally delivered infants. Research has also revealed a consistent trend in microbial variation across the microbiota in both mothers and neonates affected by GDM (Wang et al., 2018).

Nutrition profoundly influences the composition and functionality of the nascent gut microbiota, which is instrumental in fostering the evolution of a mature, adult-type microbiota. These findings highlight the importance of understanding the dynamics between the early gut microbiota and its host, emphasizing the need for further research (Bäckhed et al., 2015). Research conducted on *Macaca fuscata* revealed that maternal and postnatal high-fat diets, rather than obesity alone, influence the gut microbiota structure of offspring. Postweaning low-fat diets partially corrected the microbial dysbiosis induced by early high-fat diets. Surprisingly, high-fat diets, when introduced early, diminished the abundance of nonpathogenic *Campylobacter*, emphasizing the influence of dietary fat on the formation of symbiotic gut microbiota in primates (Ma et al., 2014).

A detailed investigation into microbiota dynamics during both pregnancy and the postpartum phase revealed a stable composition of the microbiota across various body sites during pregnancy. After childbirth, many women experience imbalances in their vaginal microbiota. These imbalances are marked by a reduction in *Lactobacillus* species and an increase in *anaerobes* such as *Prevotella*, *Peptoniphilus*, and *Anaerococcus*. Additionally, links have been identified between vaginal microbial dysbiosis, the absence of *lactobacilli*, and a greater likelihood of preterm birth (DiGiulio et al., 2015). Another study discovered a unique microbial community in the human placenta characterized by commensal taxa across various phyla. Notably, the placental microbiota most closely matches the human oral microbiome. An analysis based on 16S operational taxonomic units revealed correlations involving the placental microbiota and a history of chronic prenatal infections, such as early pregnancy urinary tract infections and preterm birth (Aagaard et al., 2014).

The establishment of early microbiota is influenced by various factors, notably the mode of delivery, diet, and nutrition. These studies highlight the importance of understanding microbiome development during infancy. They stress the need to investigate how GDM affects microbial transmission from mother to child and its impact on neonatal health. These aspects will continue to be a focal point in future studies.

#### 4.2.3 Research on the effects of probiotic supplementation

Probiotics, defined as live microorganisms that impart beneficial health effects to the host (Food and Agriculture Organization of the United Nations, World Health Organization, 2001), offer an innovative method for affecting metabolic health throughout pregnancy (Reid et al., 2013). They work by safely and effectively reshaping the gut microbiota and enhancing its functionality, aiming to counteract the harmful metabolic effects caused by pathogenic microbial communities in the gut (Clarke et al., 2012; Gomes et al., 2014). Research on the correlation between probiotics and GDM has been increasing gradually. Multiple studies have indicated that supplementing probiotics alongside dietary counseling improves glucose control in pregnant women with normal blood sugar levels (Laitinen et al., 2009). This approach has also been shown to reduce the incidence of gestational diabetes mellitus (GDM) and aid in weight management (Dolatkhah et al., 2015; Luoto et al., 2010). Additionally, probiotic supplementation results in a significant reduction in serum triglyceride and very-low-density lipoprotein (VLDL) cholesterol concentrations (Karamali et al., 2016). Another study indicated that in the treatment group receiving probiotics, significant differences were observed in the HOMA-IR and insulin levels. This group also showed notable reductions in tumor necrosis factor-alpha, interleukin-6, and high-sensitivity C-reactive protein levels (Jafarnejad et al., 2016). Therefore, probiotics may represent a promising new approach for preventing and treating glucose metabolism disorders, with potential benefits in regulating inflammation and controlling blood glucose levels (Barrett et al., 2014). Pregnant women may benefit from probiotics in maintaining serum insulin levels and potentially warding off insulin resistance (Asemi et al., 2013). In contrast, some research has shown that supplementation with the probiotic *Lactobacillus salivarius* UCC118 does not affect fasting blood glucose control, metabolic batches, or pregnancy outcomes in pregnant women. These inconsistent findings suggest that more comprehensive and authoritative evidence is needed regarding the role of probiotics in regulating blood glucose (Lindsay et al., 2015; Lindsay et al., 2014). Although there is some inconsistency in the findings, probiotic supplementation shows promise in reducing the risk of GDM and improving glucose metabolism during pregnancy. Further research is essential to identify the optimal probiotic strains and their appropriate doses to maximize these potential benefits.

#### 4.2.4 Exploration of the gut microbiota as a biomarker

Biomarkers are essential for diagnosing diseases, assessing their severity, and predicting disease progression. These findings also offer valuable insights for developing new treatments and medications.

Research has conclusively shown that among pregnant women, those diagnosed with GDM during the final trimester display a disrupted gut microbiota. In contrast, women with normal glucose levels do not exhibit these disruptions (Crusell et al., 2018). Patients with GDM have a placental microbiota with specific characteristics. Pregnant women with GDM present a diminished relative abundance of bacteria belonging to the *Pseudomonadales* order and the *Acinetobacter* genus, in contrast to those without GDM. The decreased abundance of placental *Acinetobacter* in GDM is linked to adverse metabolic markers and an inflammatory state marked by decreased blood eosinophil numbers and downregulated placental expression of IL10 and TIMP3 (Bassols et al., 2016; Kuang et al., 2017). Another study revealed that specific microbial enrichment in GDM patients was linked to blood glucose levels and highlighted the predictive potential of fecal microbial markers for GDM status. Random forest models further demonstrated the strong predictive ability of fecal MLGs, suggesting that shifts in microbial composition could identify individuals at risk of developing GDM (Kuang et al., 2017). Further investigations revealed distinct gut microbiota features in women with pregnancy-induced glucose intolerance (pGDM), alluding to the existence of exclusive microbial profiles among those predisposed to type 2 diabetes (Fugmann et al., 2015).

There are correlations between GDM and type 2 diabetes mellitus (Bao et al., 2014; Bao et al., 2015). A growing body of evidence highlights the similarity in gut microbiota dysbiosis between women with GDM and those with type 2 diabetes. Research has shown that women with GDM have a sevenfold increased risk of developing diabetes, with approximately half expected to progress to diabetes within a decade (Damm et al., 2016). Notably, distinct gut microbiota signatures can still be observed in individuals diagnosed with GDM up to 8 months postpartum (Crusell et al., 2018). Compared with normoglycemic pregnant women, GDM patients exhibit a specific vaginal and intestinal microbiome composition characterized by lower diversity (Cortez et al., 2019). These factors collectively lead to an increased risk of type 2 diabetes in women with GDM. Additionally, the identification of approximately 60,000 markers associated with type 2 diabetes has revealed that patients with this condition experience moderate dysbiosis. This dysbiosis is marked by a reduction in butyrate-producing bacteria in the gut, an increase in opportunistic pathogens, and enhanced microbial functions related to oxidative stress resistance and sulfate reduction (Qin et al., 2012). These findings indicate that the gut microbiota may serve as potential biomarkers for GDM.

Epigenetic markers include DNA methylation and (hydroxy) methylation, modifications of histones that compact chromatin, and the expression of miRNAs. Environmental factors can drive epigenetic changes, thereby positioning epigenetics as a pivotal contributor to the onset of various diseases (Elliott et al., 2019). Over the last few years, increasing epigenomic research has revealed the mechanisms through which genetic alterations affect gene expression. A previous investigation explored how epigenomics reveals the mechanisms by which maternal conditions influence the intrauterine environment and affect offspring development. The study also analyzed the potential origins of GDM (Meza-León et al., 2024). This may have a more proactive effect on the detection and treatment of GDM.

## 5 Strengths and limitations

This article provides the first systematic review of the relationship between the gut microbiota and GDM via bibliometric methods. Through comprehensive analysis and summarization via tools such as VOSviewer and CiteSpace, it presents foundational information and highlights key research trends in this field. Citespace software has certain advantages in revealing the dynamic development patterns of disciplines and identifying research frontiers within them (Chen, 2006). Moreover, VOSviewer excels in clearly presenting the relationships between disciplinary topics (Van Eck and Waltman, 2010; Van Eck and Waltman, 2014). Combining these two tools enhances the accuracy of bibliographic analysis. However, this study has several limitations. One major limitation is the exclusive reliance on data from the WoSCC database, which may not encompass all relevant sources. Future studies could consider incorporating data from multiple high-quality databases to provide a more comprehensive analysis. Second, when processing results through VOSviewer or CiteSpace, the setting of analysis parameters could lead to some of the images not providing sufficient detail, which might introduce bias into certain findings. Gaining a deeper understanding of the software’s features and learning more about the criteria for parameter selection in scientometric studies could help achieve more reliable results. Finally, we analyzed only documents in English from the database, potentially introducing a bias rooted in language limitations. This issue may be addressed if software developers enhance the capabilities to enable unified analysis across different languages, as otherwise, non-English articles could affect the accuracy of the results.

## 6 Conclusion

From a visualization standpoint, this bibliometric exploration conducted an exhaustive examination of foundational data, key research areas, and prevailing trends pertaining to the gut microbiota and GDM. The results of this study are objective and precise, offering a detailed guide for researchers currently active in or interested in this field. The analysis focused on the connection between the gut microbiota and GDM, providing valuable insights beyond those of other review articles. Various aspects, such as countries/regions, institutions, author collaborations, journals, co-cited journals, popular keywords, and burst references, were visualized. Despite ongoing research, research on the gut microbiota and GDM remains in its nascent phase, with a limited number of articles, and further investigations are needed. Given the unique nature of GDM, there is a substantial demand for safe treatment options. Lifestyle interventions and probiotic supplements are expected to remain mainstream treatment options for GDM, but research on establishing the neonatal microbiome and disease biomarkers will continue to be a focus area. With ongoing advancements in pathology research and progress in epigenetics, more effective and precise treatment methods are becoming possible.

## Data Availability

The original contributions presented in the study are included in the article/Supplementary material, further inquiries can be directed to the corresponding author/s.
