# Supplementary material for: A scientometric visualization analysis of the gut microbiota and gestational diabetes mellitus
Source: Front Microbiol. 2025 Jan 30;16:1485560. doi: 10.3389/fmicb.2025.1485560 (PMC11841407; doi:10.3389/fmicb.2025.1485560)
Supplement: Supplementary file 1 [file Data_Sheet_1.docx]

Supplementary Material

# Supplementary Figure


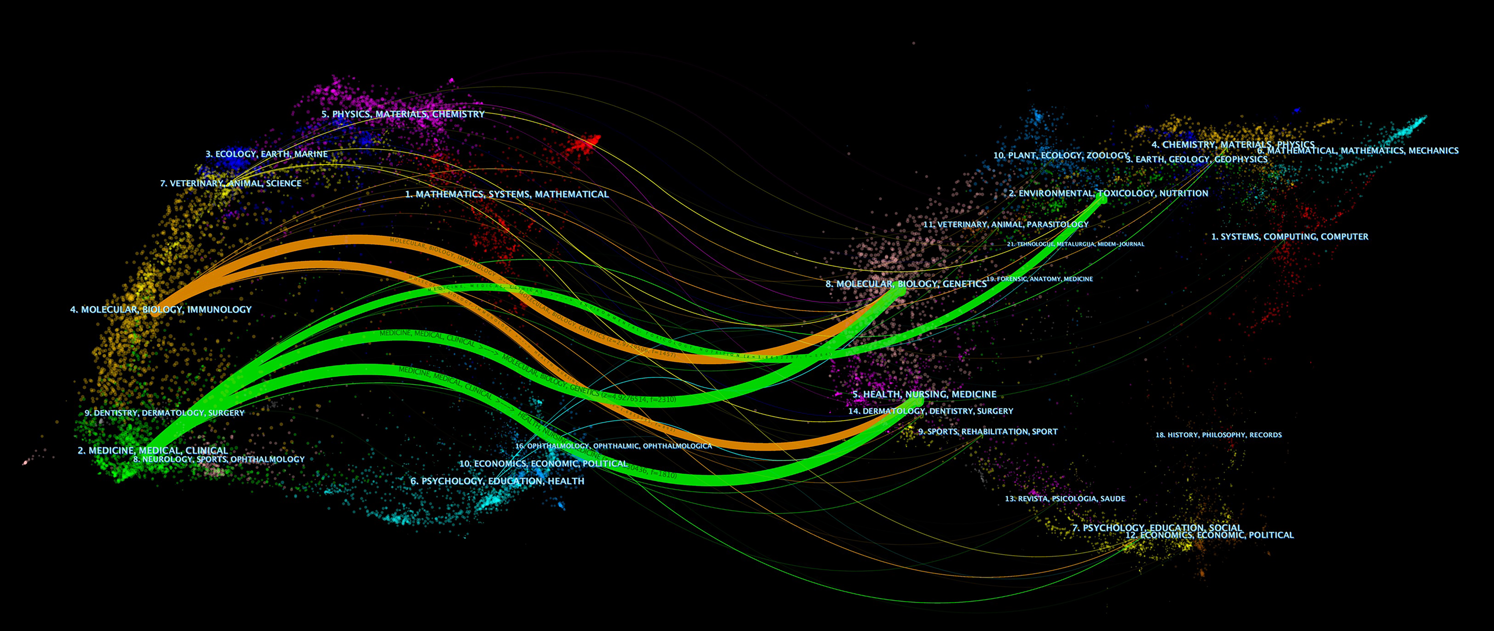


**Supplementary Figure 1.** A dual map overlay of journals in the field of gut microbiota and GDM research. On the left-hand side of the map, citing journals are shown, whereas the right-hand side features the cited journals.
